# Facile Synthesis of L-Cysteine Functionalized Graphene Quantum Dots as a Bioimaging and Photosensitive Agent

**DOI:** 10.3390/nano11081879

**Published:** 2021-07-22

**Authors:** Mila Milenković, Aleksandra Mišović, Dragana Jovanović, Ana Popović Bijelić, Gabriele Ciasca, Sabrina Romanò, Aurelio Bonasera, Marija Mojsin, Jelena Pejić, Milena Stevanović, Svetlana Jovanović

**Affiliations:** 1 “Vinča” Institute of Nuclear Sciences—National Institute of the Republic of Serbia, University of Belgrade, P.O. Box 522, 11001 Belgrade, Serbia; mila.milenkovic@vin.bg.ac.rs (M.M.); aleksandra.misovic@vin.bg.ac.rs (A.M.); draganaj@vin.bg.ac.rs (D.J.); 2 Faculty of Physical Chemistry, University of Belgrade, P.O. Box 47, 11158 Belgrade, Serbia; ana@ffh.bg.ac.rs; 3 Dipartimento di Neuroscienze, Sezione di Fisica, Università Cattolica del Sacro Cuore, 00168 Roma, Italy; Gabriele.Ciasca@unicatt.it (G.C.); sabrina.romano@unicatt.it (S.R.); 4 Fondazione Policlinico Universitario A. Gemelli IRCCS, 00168 Roma, Italy; 5 Department of Physics and Chemistry, Emilio Segrè, University of Palermo, 90128 Palermo, Italy; aurelio.bonasera@unipa.it; 6 INSTM-Palermo Research Unit, Viale delle Scienze, bdg. 17, 90128 Palermo, Italy; 7 Institute of Molecular Genetics and Genetic Engineering, University of Belgrade, 152, 11042 Belgrade, Serbia; mojsin@imgge.bg.ac.rs (M.M.); pejic@imgge.bg.ac.rs (J.P.); milenastevanovic@imgge.bg.ac.rs (M.S.); 8 Faculty of Biology, University of Belgrade, Studentski trg 16, 11000 Belgrade, Serbia; 9 Serbian Academy of Sciences and Arts, Knez Mihailova 35, 11000 Belgrade, Serbia

**Keywords:** graphene quantum dots, gamma irradiation, bioimaging, photodynamic therapy, photoluminescence, atomic force microscopy

## Abstract

Nowadays, a larger number of aggressive and corrosive chemical reagents as well as toxic solvents are used to achieve structural modification and cleaning of the final products. These lead to the production of residual, waste chemicals, which are often reactive, cancerogenic, and toxic to the environment. This study shows a new approach to the modification of graphene quantum dots (GQDs) using gamma irradiation where the usage of reagents was avoided. We achieved the incorporation of S and N atoms in the GQD structure by selecting an aqueous solution of L-cysteine as an irradiation medium. GQDs were exposed to gamma-irradiation at doses of 25, 50 and 200 kGy. After irradiation, the optical, structural, and morphological properties, as well as the possibility of their use as an agent in bioimaging and photodynamic therapy, were studied. We measured an enhanced quantum yield of photoluminescence with the highest dose of 25 kGy (21.60%). Both S- and N-functional groups were detected in all gamma-irradiated GQDs: amino, amide, thiol, and thione. Spin trap electron paramagnetic resonance showed that GQDs irradiated with 25 kGy can generate singlet oxygen upon illumination. Bioimaging on HeLa cells showed the best visibility for cells treated with GQDs irradiated with 25 kGy, while cytotoxicity was not detected after treatment of HeLa cells with gamma-irradiated GQDs.

## 1. Introduction

Graphene quantum dots (GQDs) are a zero-dimensional (0D) nanomaterial that consists of graphene sheets with π-conjugated sp^2^-hybridized carbon atoms. Since the discovery of GQDs in 2008 by Ponomarenko et al., they have attracted much attention [1]. They belong to 0D carbon nanomaterials because the movements of their excitons are confined in all three spatial directions [2]. On the basal plane and at the edges, GQDs have covalently bonded oxygen-containing functional groups [3,4]. The existence of those functional groups makes them water-soluble but also suitable for subsequent surface modification with other organic/inorganic molecules [5]. Chemical modification of GQDs has a noticeable effect on their electronic properties; depending on whether it involves the strongly electron-accepting or electron-donating molecules [6].

GQDs are semiconductors with a lateral size below 100 nm and a tunable band gap, usually between 1 and 4 eV [7,8], while graphene itself is a semi-metal with a 0 band gap [9]. The non-zero band gap is a consequence of quantum confinement and the edge effect in GQDs [10,11,12]. With the size and surface chemistry modification, the band gap can be tuned [12,13]. Thus, GQDs show stable photoluminescence in the visible part of the spectrum [12].

Other properties of GQDs are chemical inertness, low cytotoxicity, biocompatibility and good dispersibility in water [11,12,14,15,16,17]. The non-toxic nature and excellent photostability distinguish them from other inorganic quantum dots [18]. After administration, a few ways of GQDs elimination from the animal organisms were found [19,20,21]. Smaller size GQDs excreted from the mice organism, predominantly through the kidney and urine, while larger ones are mainly eliminated through the liver. Thanks to these mechanisms, over-accumulation in tissues and organs is avoided. The above-mentioned properties make GQDs a good candidate for biomedical applications such as bioimaging and photodynamic therapy [3,22].

One of the methods for GQDs synthesis is electrochemical oxidation. This simple and low-price procedure is based on oxidation and exfoliation of graphite rods, immersed in the electrolyte solution [23,24,25]. A high redox potential produces radicals OH• and H• from water which create the pressure between graphene layers. This pressure leads to gaps in van der Waals interactions and results in the formation of defects in graphene planes [26]. Both exfoliation and electrochemical cutting of graphite rods cause the formation of nanometer-scale structures—graphene quantum dots [27]. Depending on the employed electrolyte, the obtained GQDs have different surface functionalization, degree of oxygenation and, consequently, photoluminescence quantum yields [28,29].

Gamma irradiation is a possible approach to the structural modification of carbon nanomaterials [30]. It is a simple and clean method for altering the structure, morphology and physical–chemical properties of carbon nanomaterials. The effects of irradiation strongly depend on material type, irradiation dose, and medium [30]. The mediums used for irradiation of GQDs were deionized water, a mixture of deionized water and isopropyl alcohol (IPA), ethanol, and ethylenediamine (EDA) [31,32,33]. By changing the medium, it is possible to achieve a reductive or oxidative environment during irradiation. For example, Chan et al. irradiated graphene oxide (GO) in water and a water–IPA mixture with doses of 115.2, 230.4 and 345.6 kGy [34]. XPS spectra showed that after irradiation, the amount of oxygen-containing functional groups was significantly reduced in the presence of IPA; the C/O ratio was increased from 1.18 for 0 kGy to 9.98 for an irradiation dose of 345.6 kGy. These results proved that IPA created a reductive environment. Another study showed that carbon quantum dots (CQDs) irradiated with doses from 0 to 15 kGy in the presence of IPA or isobutanol and purged with N_2_ showed more hydroxyl groups compared to the reaction performed in the presence of only water [35]. Jovanović et al. showed that gamma irradiation in oxidative mediums (H_2_O, NH_4_OH) can cause the shortening, unbundling and annihilation of single-wall carbon nanotubes (SWCNTs) [36]. Consequently, the reductive mediums (H_2_O and NH_4_OH both mixed with isopropyl alcohol) eliminated C–O groups and increased the fraction of sp^2^-hybridized carbon atoms in the SWCNTs structure. For the first time, GQDs were gamma-irradiated in 2015 [31]. The medium for irradiation was a mixture of water and IPA (4 *v*/*v*%), while the applied doses of gamma irradiation were 20, 50, 100 and 200 kGy. This study showed that GQDs’ properties such as diameter, photoluminescence intensity, band gap, and singlet oxygen production differ with the change in the irradiation dose. The main improvements in properties were found in the sample irradiated with a dose of 50 kGy: the average diameter decreased from 24 to 18 nm, the oxygen content increased from 35.9 to 45.1 wt.%, photoluminescence (PL) intensity was 4.5 times higher, and the highest production of singlet oxygen upon the UV illumination was detected [31].

Until now, there have been reported a few top-down syntheses of N,S-doped GQDs. Ouyang et al. reported top-down hydrothermal synthesis from graphite as the carbon source and thiourea as a heteroatom dopant [37]. The study showed that the incorporation of S- and N-atoms reduces the number of graphene layers in GQDs from 4–6 to 1–2. XPS spectra revealed the successful doping of heteroatoms in a GQD structure and the PL intensity at an excitation wavelength of 320 nm was the highest for the sample synthesized in the 1:1 ratio of graphene and thiourea [37]. Another top-down procedure was developed by Xu et al. where mesoporous polythiophene-derived carbon was used as a starting material [38]. With the temperature increase, the nitrogen and sulfur contents decreased from 3.7 to 2.2 at% and 8.6 to 8.0 at%, respectively. The oxygen content was increased from 40 to 43 at%. Zhang et al. produced N-GQDs and N,S-GQDs and investigated how the optical properties of GQDs changed with the introduction of sulfur [39]. With S doping, the average radius increased from 3.16 to 5.25 nm, while the PL enhanced as well as the quantum yield from 10.1 to 18.6%. Both the O- and N-content was enriched, which indicates that S-doping enables the formation of more reactive graphene sites where N or O can easily bind.

Here, we reported a one-step functionalization of GQDs by gamma irradiation in the presence of IPA as an oxygen radical scavenger and amino acid L-cysteine as a S, N- heteroatom donor. Before irradiation, samples were purged with Ar gas. Due to the presence of IPA, cysteine and the removal of oxygen by Ar gas, the highest concentration of H_2_ can be achieved [40]. These conditions offer the reductive environment for irradiation and the source of N and S atoms. Amino groups can be introduced in the GQDs structure using the organic chemistry reactions such as treatment with a combination of N-(3-Dimethylaminopropyl)-N′-ethylcarbodiimide (EDC) and N-hydroxysuccinimide (NHS) [41,42], acid chloride formation [43], carbamate or imine formation [44], etc., or by hydrothermal treatment with ammonia or sulfur [45,46]. These reactions demand the use of reactive and aggressive chemicals, and they are often time-consuming. The method proposed in this paper does not use hazardous chemicals and it took place in only one synthetic phase; heteroatoms were incorporated.

## 2. Materials and Methods

### 2.1. Synthesis of Pristine GQDs and Gamma-Irradiated GQDs

Graphene quantum dots were synthesized by using electrochemical oxidation of graphite electrodes purchased from Ringsdorff-Werke GmbH (Bonn, Germany), with 99.999% purity and a diameter of 3.05 mm. Both electrodes, anode and cathode, were graphite ones. Electrodes were washed with Milli-Q water and ethanol (96 *v*/*v*%). Then, they were dipped in the electrolyte which was prepared by sonication of a NaOH (3 g) in 96 *v*/*v*% ethanol (100 mL). Applied current intensity was 20 mA and a voltage was set at 20 V. After 8 h, the color of dispersion changed from light yellow to dark brown, which indicated the formation of GQDs. After that, ethanol was removed with evaporation at a reduced pressure. Obtained as-synthetized material was redispersed in water in a concentration of 3 mg mL^−1^ and placed into a dialysis bag with molecular weight cut-off (MWCO) of 3.5 kDa. We monitored the pH values of the dispersion all the time. The dialysis was complete when pH remained stable at a value of 7 even after changing the water. In this way, the residual amount of NaOH was removed from the GQDs solution. Water from dispersion was then evaporated by heating at 80 °C to obtain the powder GQDs which were labeled as pristine Graphene Quantum Dots (p-GQDs).

To synthesize modified GQDs, p-GQDs were exposed to gamma irradiation. Samples for irradiation were prepared by sonication of GQDs in Milli-Q water at a concentration of 1 mg mL^−1^, with 2 wt.% L-cysteine and 1 *v*/*v*% isopropyl alcohol (IPA). Then, the mixture was purged with Ar gas for 15 min and exposed to gamma irradiation using Co-60 as an irradiation source. Samples were exposed to different doses of irradiation: 25, 50 and 200 kGy. Irradiated samples were dialyzed in the bags with MWCO of 3.5 kDa and evaporated to dryness. The obtained powders were collected and used for further characterizations. Samples were labeled as GQD-cys-25, GQD-cys-50, and GQD-cys-200 which corresponds to applied irradiation doses of 25, 50 and 200 kGy, respectively.

### 2.2. Methods

#### 2.2.1. Ultraviolet–Visible Spectroscopy

Absorption measurements were recorded on a GBC Cintra 6 spectrophotometer (GBC Dandenong, Australia) using quartz cell with 1 cm path length and 4 mL volume. UV–Vis spectra were recorded from 200 to 800 nm. For these measurements, GQDs were sonicated in demineralized water for 30 min. The concentration of produced dispersion was 0.25 mg mL^−1^. Measurements were conducted in the air environment, at room temperature.

#### 2.2.2. Photoluminescence Spectroscopy

Photoluminescence spectra (PL) were recorded on a HORIBA Jobin Yvon FluoroMax-4 spectrometer (HORIBA, Kyoto, Japan). All of the GQD samples were dispersed in methanol at a concentration of 0.025 mg mL^−1^. Excitation wavelengths for obtaining PL spectra were in the range of 300–400 nm. All measurements were conducted in the air environment at room temperature using a quartz cuvette with a path length of 1 cm and a volume of 4 mL. Fluorescence quantum yields (QY) were calculated using the Equation (1):

QY_GQDs_ = QY_REF_(A_REF_/A_GQDs_)(F_GQDs_/F_REF_)(n_GQDs_/n_REF_)^2^ (1)

where QY is fluorescence quantum yield, A is absorption, F the integrated fluorescence intensity of the emitted light and n is the index of refraction of the solvent. The subscript “REF” denotes the reference sample of Rhodamine B, QY = 31% and subscript “GQD” refers to the GQD samples.

#### 2.2.3. Atomic Force Microscopy

Atomic Force Microscopy (AFM) measurements were carried out using a Quesant microscope (Agoura Hills, CA, United States), which was operating in a tapping mode. For non-contact high-frequency applications, we used a rotated monolithic silicon probe Q-WM300, with standard silicon tips (NanoAndMore GmbH, Wetzlar, Germany) and a force constant of 40 N m^−1^. Aqueous dispersions of GQDs in a concentration of 0.25 mg mL^−1^ were sonicated for 30 min and deposited on mica substrate by spin-coating at 3500 rpm for 1 min. Gwyddion software was used to analyze the lateral size and height of the GQDs. For diameter distribution histograms, we analyzed around 1000 particles, and for height distribution histograms we analyzed more than 100 particles.

#### 2.2.4. Dynamic Light Scattering

The particle size distribution was determined using the dynamic light scattering (DLS) technique (Malvern, Herrenberg, Germany). This system was equipped with a 633 nm helium–neon laser as a light source and measured the particle size at a scattering angle of 173°. All measurements were conducted at a controlled temperature of 20 °C and fixed positions (4.65 nm) with an automatic attenuator.

#### 2.2.5. Scanning Electron Microscopy

Scanning electron microscopy (SEM) with energy-dispersive X-ray (EDS) spectroscopy was conducted using an FEI ESEM Quanta 200 microscope (FEI Company, Hillsboro, OR, Unites States). Samples p-GQDs, GQDs-cys-25 and GQDs-cys-200 were prepared by dissolving the pristine powders in ethanol; the so-prepared solutions were drop-casted onto clean Si-substrates (10 mm × 10 mm), and finally the samples were dried under vacuum overnight. For SEM imaging, only sample GQDs-cys-50 was deposited onto Al support using the same procedure. Samples were scanned at the SEM microscope without any further preparation (no metallization), and using the “low vacuum mode”. Energy-dispersive X-ray (EDS) measurements were obtained focusing the analysis over an area of ca. 230 × 200 microns. All samples were deposited on Si support. Data analysis was conducted using the EDAX Genesis EDS microanalysis software (AMETEK, Inc., Berwyn, PA, Unites States).

#### 2.2.6. Fourier-Transform Infrared Spectroscopy

Fourier-Transform Infrared (FTIR) spectra were acquired using the Thermo Scientific Nicolet 6700 FTIR instrument (Thermo Fischer Scientific, Waltham, MA, United States) in attenuated total reflection (ATR) mode. The spectral range was from 900 to 4000 cm^−1^. All of the GQDs samples were in powder form.

#### 2.2.7. Electron Paramagnetic Resonance Spectroscopy

The electron paramagnetic resonance (EPR) spectra were recorded using a Bruker BioSpin ELEXSYS-II E540 EPR spectrometer with the following experimental parameters: microwave frequency 9.85 GHz (X-band), microwave power 10 mW, modulation amplitude 1 G, modulation frequency 100 kHz and a sweep time of 60 s. 2,2,6,6-tetramethylpiperidine (TEMP) was used as a spin trap agent. Generation of ^1^O_2_ was measured in GQDs before and after gamma irradiation, where air saturated dispersions were mixed with TEMP and exposed to light. The spin trap method is based on the chemical reaction between TEMP and ^1^O_2_, which forms a stable radical adduct, (2,2,6,6-tetramethylpiperidin-1-yl)oxyl (TEMP-^1^O_2_ or TEMPO). TEMPO shows a triplet signal in the EPR spectrum. To investigate the ability of GQDs to produce singlet oxygen under illumination, samples were dispersed in ethanol in a final concentration of 0.2 wt.%. Then, TEMP was added in a concentration of 30 mM and mixtures were purged with air. These mixtures were exposed to blue light (λ = 360–400 nm) for 100 min. EPR spectra were recorded before and during light exposure. Additionally, GQDs were incubated with TEMPO (3 mM) and after illumination in the same condition, EPR was recorded.

#### 2.2.8. Bioimaging

Aqueous solutions of p-GQDs, GQD-cys-25, GQD-cys-50, and GQD-cys-200 were mixed with cell culture media and added to the cells in the final concentration of 100 µg mL^−1^. Untreated HeLa cells were used as control. After 24 h of incubation, the medium was removed, cells were washed three times in PBS and subjected to optical imaging. Cellular uptake of p-GQDs, GQD-cys-25, GQD-cys-50, and GQD-cys-200 was monitored using Olympus BX51 fluorescence microscope Olympus (Olympus, Tokyo, Japan) with Spectrum Aqua filter. Images were captured at 10x and 20x objectives and analyzed using the CytoVision 3.1 software (Applied Imaging Corporation, Santa Clara, CA, United States).

#### 2.2.9. MTT Assay

For (3-(4,5-Dimethylthiazol-2-yl)-2,5-Diphenyltetrazolium Bromide) cell viability assay (MTT) assay, HeLa cells were seeded at concentration 1 × 10^4^ cells/well in 96-well plates and allowed to attach overnight. The following day, cells were treated with GQDs (p-GQDs, GQDs-cys-25, GQDs-cys-50, and GQDs-cys-200) at concentrations of 1, 10, 25, 50, and 100 μg mL^−1^ for 24 and 48 h. At the indicated time points, medium was removed, cells were washed three times with phosphate-buffered saline and cell viability was determined using MTT assay. Cells were incubated for 1 h in MTT solution, 0.5 mg mL^−1^ cell culture media, (Merck KGaA, Gernsheim, Germany), formed formazan crystals were dissolved in DMSO (dimethyl sulfoxide) (SERVA Electrophoresis GmbH, Heidelberg, Germany) and absorbance was measured at 540 nm using an Epoch Microplate Spectrophotometer (BioTek, VT, USA). All experiments were performed in triplicates, repeated at least three times. The relative viability of the treated cells was calculated as a percentage of the vehicle control (HeLa cells treated with methanol) set to 100%. Data were analyzed using SPSS Statistics 28 (IBM, Armonk, NY, United States). Due to the technical issues we only obtained data from one treatment with GQDs-cys-50 at concentration 100 μg mL^−1^ at 24 h time point. Accordingly the mean, standard deviation and statistical significance were not calculated for this point.

## 3. Results and Discussion

GQDs are produced using an electrochemical approach. Two graphite rods were immersed in 3% NaOH in ethanol and the current was applied. After 8 h, the dispersion with NaOH, ethanol and GQDs was collected. To isolate dots, ethanol was removed by evaporation, while NaOH was eliminated in the process of dialysis. To achieve a one-step modification of GQDs, they were dispersed in a water–IPA mixture and L-cysteine was added. This mixture was purged with Ar and irradiated with doses from 25 to 200 kGy. After isolation in the process of dialysis, gamma-irradiated GQDs were characterized and these results are presented in the following text.

### 3.1. UV–Vis Spectroscopy

**Figure 1:**
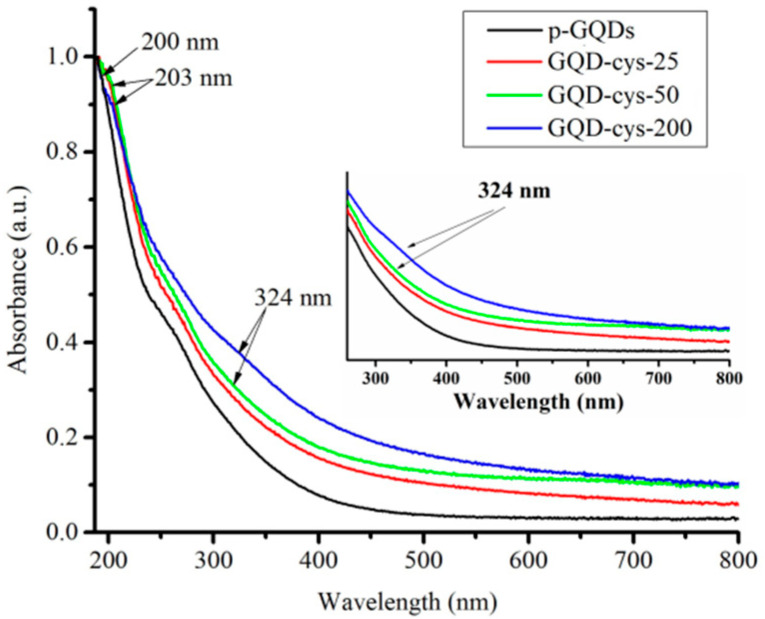
UV–Vis spectra of p-GQDs (black), GQD-cys-25 (red), GQD-cys-50 (green), GQD-cys-200 (blue).

The optical properties of pristine and gamma-irradiated GQDs were determined by UV–Vis spectroscopy and the obtained spectra are presented in Figure 1.

As we can see, all GQDs samples showed typical absorption peaks in the UV region. For p-GQDs, the main absorption band at around 200 nm can be attributed to π–π* transitions from aromatic the C–C bond [37,47]. In the case of gamma-irradiated GQDs, this band showed a small shift to 203 nm for gamma-irradiated samples. This can be assigned to the changes in the aromatic structure and modification of GQDs [32]. For GQD-cys-200 and GQD-cys-50, the peak can be observed in a wavelength of about 324 nm. It stems from a carboxyl functional group and n–π* transition of C=O groups [48].

### 3.2. PL Spectroscopy

**Figure 2:**
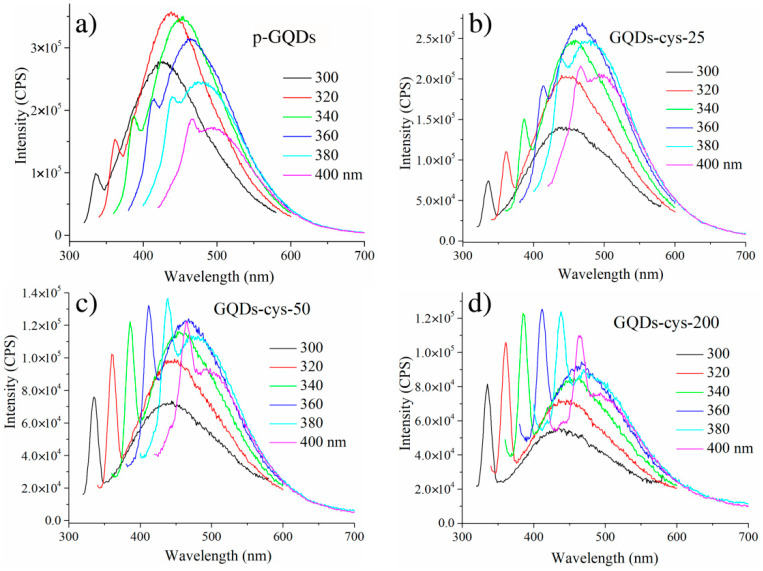
Emission spectra of p-GQDs (**a**), GQDs-cys-25 (**b**), GQDs-cys-50 (**c**) and GQDs-cys-200 (**d**). The excitation wavelengths were between 300 and 400 nm.

In Figure 2, PL emission spectra of pristine and gamma-irradiated GQDs are presented. For the excitation, we used wavelengths between 300 and 400 nm. All samples showed the shift in the center of emission upon different excitation wavelengths. This kind of optical behavior is named excitation-dependent and it is very often observed in GQDs [49].

Colloidal dispersions of GQDs showed a broad PL emission (Figure 2). The center of the emission for p-GQDs was shifted from indigo at 426 nm (2.91 eV) to a blue color, to 494 nm (2.51 eV) upon excitation from 300 to 400 nm, respectively. The position of emission bands for all samples is presented in Table 1. In the case of gamma-irradiated dots, they showed a mostly blue emission, in the range of 440–495 nm. The position of the emission band shifted with gamma irradiation toward higher wavelengths, as presented in Table 1. However, between doses of 25, 50 and 200 kGy, there were no significant differences between the positions of these bands.

PL emission is associated with Cπ*→Cπ, Nπ*→Cπ and Oπ*→Cπ transitions for N-doped GQDs [50]. Due to various possible transitions, the emission bands are broad. Photoluminescence of GQDs stems from both the graphene core and functional groups [51]. While graphene is responsible for the intrinsic emission, the surface state emission is controlled by groups. Blue photoluminescence of GQDs is associated with hydroxyl functional groups, while green is related to carboxyl and amide functional groups [52,53]. Gamma irradiation causes changes in the structure of GQDs which affecting the PL properties by tuning them [31,32]. One more difference between the emission spectra of p-GQDs and gamma-irradiated dots is that the highest intensity of the emission was detected for p-GQDs at the excitation wavelength of 320 nm, while in the case of gamma-irradiated dots, the highest intensity of the main emission band was detected at the excitation wavelength of 360 nm.

Photoluminescence QYs of gamma-irradiated GQDs were calculated using Rhodamine B as a reference and these results are presented in Table 2. The highest PL QY was detected after gamma irradiation at a dose of 25 kGy, while for doses of 50 and 200 kGy, QYs were only 5.15 and 3.12%, respectively. Our previous study showed that PL QY for p-GQDs was only 1.45% at the excitation wavelength of 340 nm [32]. These results suggest that gamma irradiation improved PL QY of GQDs and that the largest improvement was achieved at a dose of 25 kGy.

### 3.3. AFM Microscopy

**Figure 3:**
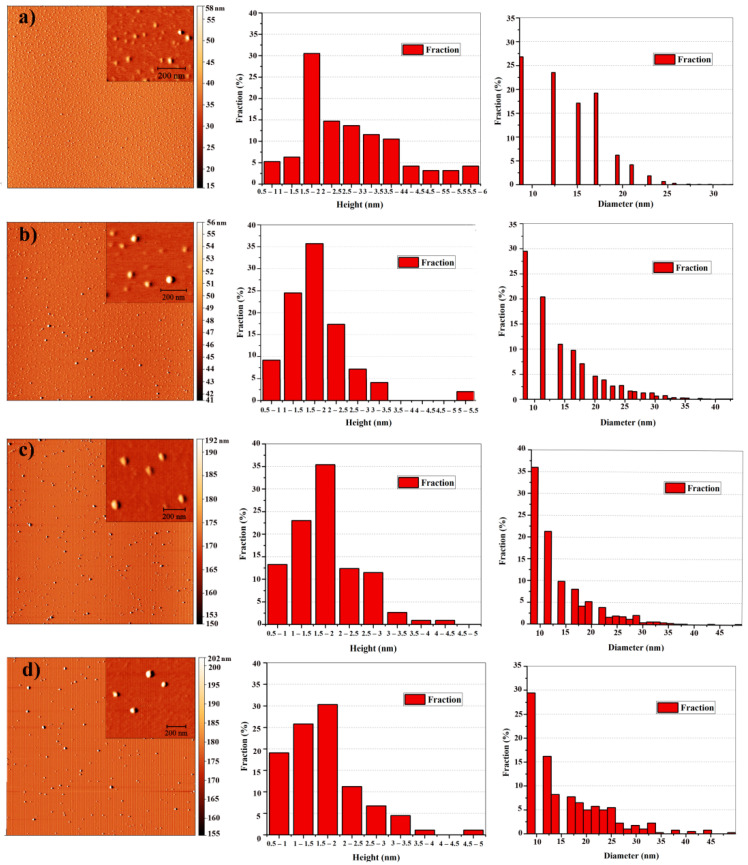
AFM images and histograms of height and diameter distribution for p-GQDs (**a**), GQD-cys-25 (**b**), GQDs-cys-50 (**c**), and GQD-cys-200 (**d**).

The morphology, height and diameter distribution of p- and gamma-irradiated GQDs were measured with an AFM microscope. The obtained AFM images and histograms of the height and diameter distribution are shown in Figure 3. The fraction of GQDs is a percentage of dots that are a certain height or diameter (the number of dots divided with the total number of analyzed GQDs).

All images show well-dispersed, uniform, round-shaped particles without large agglomerates. From the height distribution histogram of all samples, it can be seen that the height of GQDs was between 0.5 and 2.5 nm. Since the thickness of one graphene layer measured by AFM was between 0.7 and 1 nm [4], it can be stated that most of GQDs consist of one to four graphene layers. While p-GQDs have a smaller percentage of single and double-layered GQDs (5.26% and 6.31%, respectively), irradiated GQDs are mostly multilayered with a higher percentage of single and double graphene layers (9.2% and 24.5%; 13.3% and 23.0%; 19.1% and 25.8% for GQD-cys-25, GQD-cys-50 and GQD-cys-200, respectively). This result indicates that gamma irradiation increases the level of layer separation [31]. The average particle diameter was in a range of 20 to 30 nm after AFM tip deconvolution. With a higher irradiation dose, the particle diameter also increased. Accordingly, the p-GQDs diameter calculated from tip deconvolution was 20.4 nm, and for GQD-cys-25, GQD-cys-50 and GQD-cys-200 the diameter was 25.5 nm, 28.0 nm and 29.0 nm, respectively (Table 3).

### 3.4. DLS Analysis

**Figure 4:**
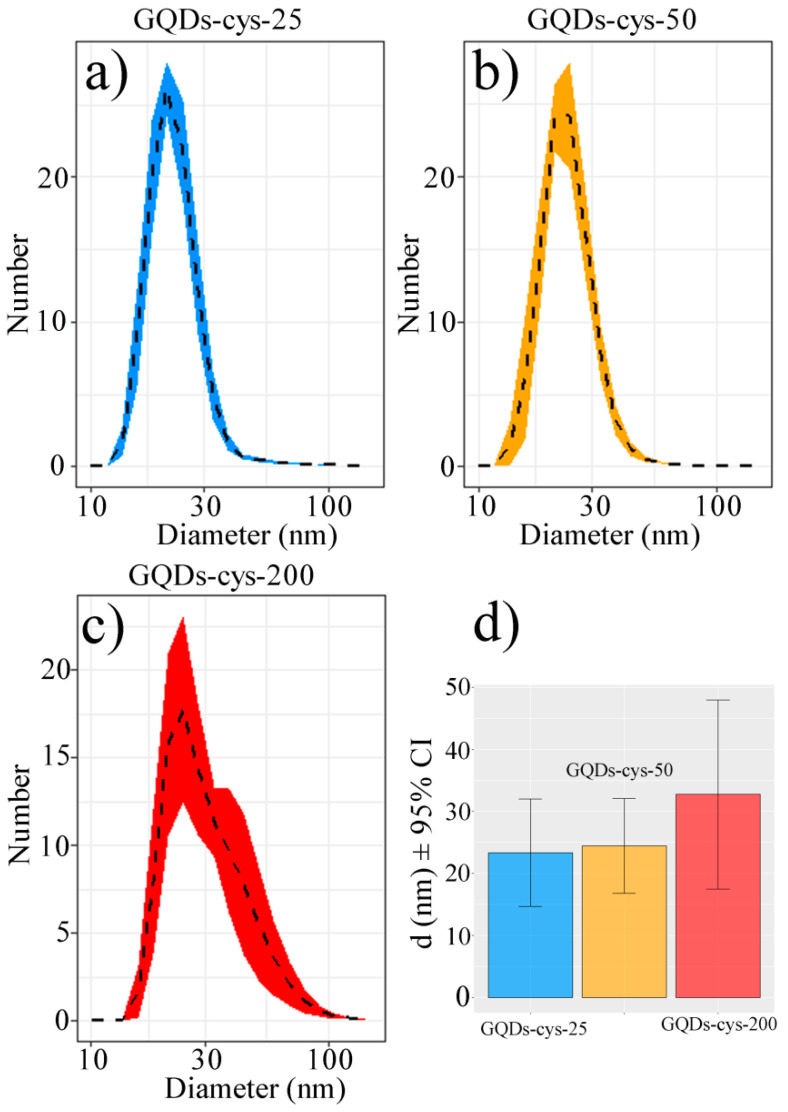
Average number-weighted diameter distribution function for GQD-cys-25 (**a**), GQD-cys-50 (**b**), GQD-cys-200 (**c**) and mean diameter (**d**).

The effects of surface functionalization on the size distribution in gamma-irradiated GQDs were investigated by using DLS. The obtained number-weighted average diameter distribution function is presented in Figure 4a–c.

The results show that both GQD-cys-25 and GQD-cys-50 had a similar shape of the diameter distribution function, while GQD-cys-200 was strongly right-skewed, with a heavy right tail. The average hydrodynamic diameter was approximately 23, 24 and 27 nm for GQD-cys-25, GQD-cys-50 and GQD-cys-200, respectively (Figure 4d). The following average PDI values were measured: 0.37 (GQD-cys-25), 0.35 (GQD-cys-50), and 0.23 (GQD-cys-200). These results are in agreement with AFM measurements. As we previously reported for p-GQDs, the average hydrodynamic diameter was 13.85 nm [32].

### 3.5. SEM-EDS

**Figure 5:**
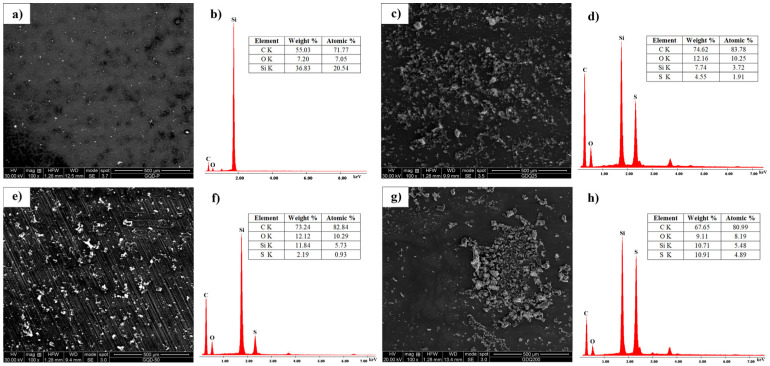
SEM images and EDS spectra of p-GQDs (**a**,**b**), GQD-cys-25 (**c**,**d**); GQD-cys-50 (**e**,**f**) and GQD-cys-200 (**g**,**h**).

The surface morphology and chemical composition of irradiated GQD-cys samples were determined by SEM-EDS analysis. The obtained results are presented in Figure 5.

From SEM images (Figure 5a,c,d,g), it can be seen that the surface morphology of all analyzed samples was similar, with plate-like structures and agglomerates. The elemental content was investigated using EDS. The obtained results (Figure 5b,d,f,h) showed that p-GQDs had only C and O atoms in their structure, while GQD-cys samples were mainly composed of C, O and S atoms. This indicates that after irradiation, C and O atoms were still present in the sample. Silicon was detected in each EDS spectrum from the substrate used in the experiment. The atomic percentage of C and O was similar for GQD-cys-25 (C 83.78 at%; O 10.25 at%) and GQD-cys-50 (C 82.84 at%; O 10.29 at%). With the increase in the irradiation dose, the content of C and O was lowered for GQD-cys-200 (80.99 at% for C and 8.19 at% for O atoms). This indicates the decrease in oxygen-containing functional groups over the surface of GQD-cys. Sulfur was also found in each of these samples in the amount of 1.91 at%, 0.93 at% and 4.89 at% for GQD-cys-25, GQD-cys-50 and GQD-cys-200, respectively. These results confirmed the successful S-doping of GQDs and the largest level was detected at a dose of 200 kGy.

### 3.6. FTIR Spectroscopy

**Figure 6:**
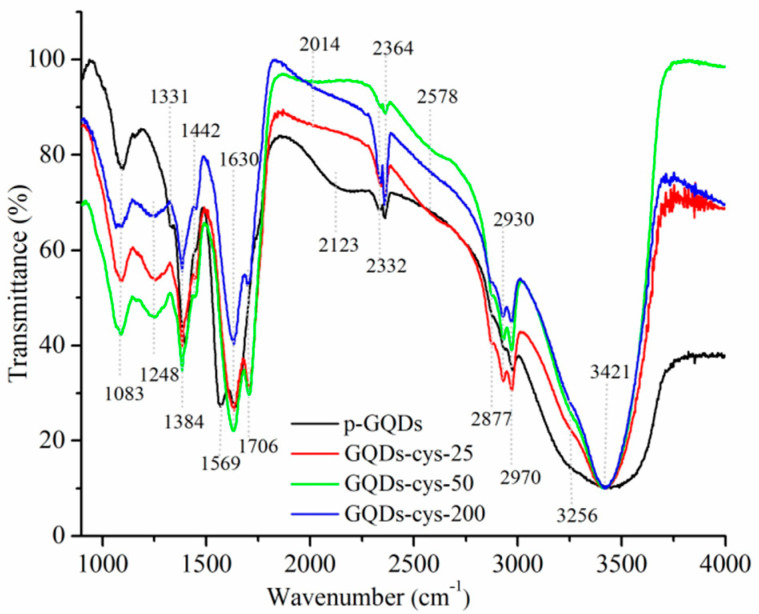
FTIR spectra of p-GQD (black), GQD-cys-25 (red), GQD-cys-50 (green) and GQD-cys-200 (blue).

To investigate the structure and identify functional groups on the surface of GQD-cys, an FTIR analysis was employed. The obtained results are presented in Figure 6.

The strong and broad absorption band at 3421 cm^−1^ is attributed to the stretching vibration of OH groups [54]. Unlike p-GQDs, all irradiated GQD-cys showed a peak at approximately 3256 cm^−1^ which corresponds to N–H stretches. These results confirmed the presence of N atoms on the surface of GQD-cys. Three low-intensity bands at around 2970 cm^−1^, 2930 cm^−1^ and 2877 cm^−1^ originated from vibrations of sp^2^- and sp^3^-CH groups [8,32]. Next, the weak signal at 2578 cm^−1^, found only in the gamma-irradiated FTIR spectra, originated from the vibration of the S–H bond [55,56]. The peaks at 2364 and 2332 cm^−1^ are related to the background CO_2_.

The broad and characteristic band for GQD-cys with peaked at approximately 1630 cm^−1^ and 1706 cm^−1^ stemmed from C=O stretching vibrations in COOH and the amid-carbonyl (–NH–CO–) group, respectively [56,57]. The signal of amid-carbonyl vibrations was formed due to the reaction of amide and carboxylic groups. The spectral band at around 1630 cm^−1^ also proves that the aromatic structure of GQD-cys was preserved after gamma irradiation [56]. For p-GQDs, peaks showed at around 1570 cm^−1^ and 1630 cm^−1^ stems from C=C and C=O stretching vibrations [8,58]. The peak at 1384 cm^−1^ was present in all samples, and it is the result of C–OH bending vibrations [59]. The intensity of this band was lower for gamma-irradiated samples due to the replacement of oxygen-containing functional groups with S and N functional groups. The band at 1250 cm^−1^ appeared only in gamma-irradiated samples and it is assigned to the C=S stretching vibrations from the thiocarbonyl groups [58]. This band indicates the substitution of oxygen in the carbonyl or carboxylic group with sulfur, thus, achieving the successful binding of S atoms in the GQDs structure. Finally, the peak at 1083 cm^−1^ could be seen in all analyzed samples, and it stemmed from the vibration of C–O bonds.

FTIR spectroscopy confirmed the doping of gamma-irradiated GQDs with S- atoms as it was observed by SEM-EDS spectroscopy. Additionally, the successful incorporation of N- atoms in the structure of GQDs was detected. These results proved that gamma irradiation in the presence of L-cysteine and IPA in a reduction atmosphere leads to S,N- doping of GQDs.

### 3.7. EPR Spectroscopy with ^1^O_2_ Trap

**Figure 7:**
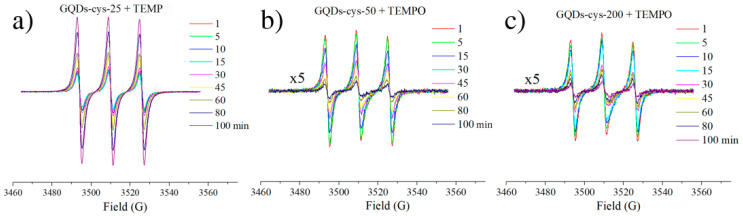
EPR spectra of light illuminated (1–100 min) GQD-cys-25 incubated with 30 mM TEMP (**a**), and GQD-cys-50 (**b**), and GQD-cys-200 (**c**) incubated with 3 mM TEMPO.

EPR spectroscopy was used to investigate the ability of GQDs to produce singlet oxygen upon light illumination (360–400 nm), using TEMP as a selective ^1^O_2_ trap. In the reaction with singlet oxygen, the EPR silent TEMP is converted to the EPR-active TEMPO radical, characterized by an isotropic three-line signal [60]. The results showed that only the GQD-cys-25 dispersion can produce singlet oxygen when it is exposed to light (no radical formation is observed in the absence of light). The radical production was followed for 100 min, during which it was observed that the TEMPO signal increased with illumination time (Figure 7a). Since the TEMPO radical was not detected upon the illumination of the GQD-cys-50 and GQDs-cys-200 dispersions in the presence of TEMP, they were further investigated. Interestingly, when these dispersions were incubated with TEMPO, and subsequently exposed to light up to 100 min, a reduction in the EPR signal was observed (Figure 7b,c). This may suggest that the light-activated GQD-cys-50 and GQDs-cys-200 dispersions are strong oxidizing agents which convert the TEMPO radical into its EPR inactive oxoammonium cation form. Therefore, it is most likely that these GQDs are not incapable of singlet oxygen production, but rather that they produce ^1^O_2_ to a greater extent than GQD-cys-25.

### 3.8. Bioimaging

**Figure 8:**
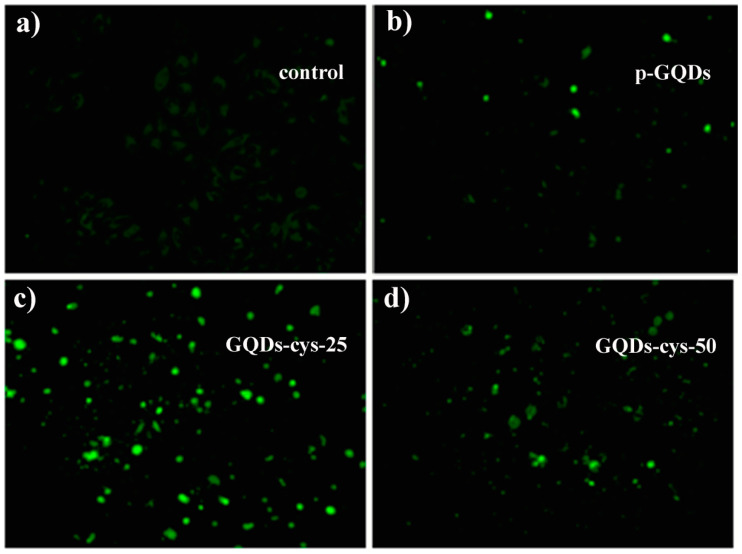
Fluorescence microscope images of HeLa cells without GQDs (**a**), cells treated with p-GQDs (**b**), with GQD-cys-25 (**c**), and GQD-cys-50 (**d**). HeLa cells were exposed to GQD samples at a concentration of 100 µg mL^−1^ and incubated for 24 h.

The cellular uptake of p-GQDs, GQD-cys-25, GQD-cys-50 and GQDs-cys-200 into HeLa cells was analyzed using fluorescent microscopy and results are presented in Figure 8. All tested quantum dots (p-GQDs, GQD-cys-25, GQD-cys-50, and GQDs-cys-200) entered into HeLa cells (Figure 8a–d). The best visibility of HeLa cells was achieved when the cells were treated with GQDs-cys-25 (Figure 8c). The calculation of PL QY showed the highest values for the GQD-cys-25, while the size of all samples was similar (14–30 nm). It was found that GQDs with a size below 100 nm, entered the cell through the passive mechanism [61,62]. However, the difference in the visibility of the cells stems from the superior photoluminescence of GQDs-cys-25. Thus, this sample is considered the best candidate as a bioimaging agent.

### 3.9. Cytotoxicity

**Figure 9:**
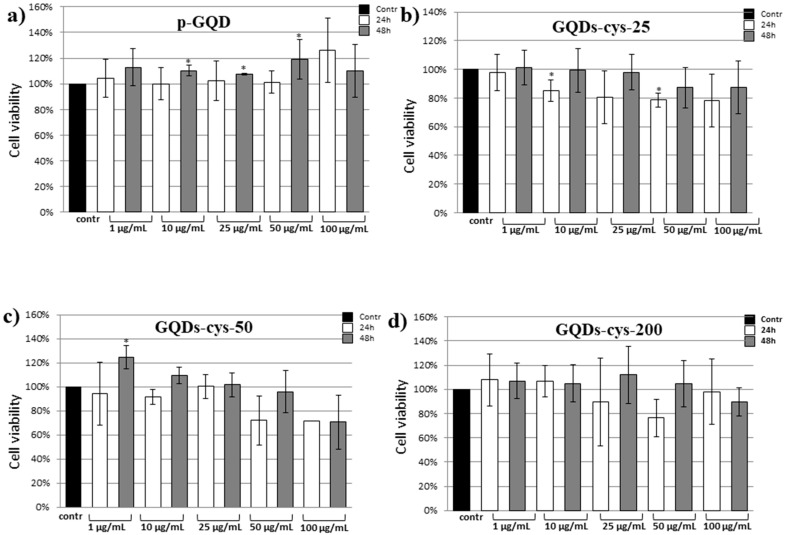
Cytotoxic activity of GQDs against HeLa cells: p-GQDs (**a**), GQDs-cys-25 (**b**), GQDs-cys-50 (**c**), and GQDs-cys-200 (**d**). Cells were treated for 24 and 48 h with increasing concentrations (1, 10, 25, 50, and 100 µg mL^−1^) of the tested GQDs samples. Cell viability was expressed as the percentage of absorbance relative to the vehicle control (HeLa cells treated with methanol), which was set at 100%. Data are presented as the mean ± SD of at least three independent experiments. Asterisk denotes a significant difference from the control (* *p* < 0.05). SD—standard deviation.

To estimate how safe it is to use materials in bioimaging, one of the first tests is cytotoxicity. However, agents for photodynamic therapy must also be non-toxic in the absence of light. Thus, the cytotoxicity of both p-GQDs and gamma-irradiated GQDs was evaluated on the HeLa cells (Figure 9) using an MTT assay. The effects of both GQD concentrations (1–100 μg mL^−1^) as well as exposure time (24–48 h) on the cell viability were studied. All measurements were conducted in dark, to avoid photo-induced toxicity. The results of the MTT assay showed that p-GQDs were not considered toxic toward HeLa cells at concentrations up to 100 μg mL^−1^ (Figure 9). As shown in all gamma-irradiated GQDs, either cytotoxicity (cell viability between 80 and 100%) nor very low cytotoxicity (cell viability slightly below 80%) was detected.

## 4. Conclusions

In this study, we exposed a water dispersion of GQDs mixed with L-cysteine (2 wt.%), and IPA 1 *v*/*v*% to gamma irradiation at doses of 25, 50 and 200 kGy. Dispersions were purged with Ar gas to remove oxygen. By selecting these conditions we were able to achieve reductive conditions, but also the bonding of N and S functional groups in the GQD structure. The largest improvement of PL properties was detected for dots irradiated at 25 kGy where QY was calculated to be 21.65%, while for 50 and 200 kGy, this parameter was lower (5.15 and 3.12%, respectively). SEM-EDS analysis indicated that S atoms were present in GQDs. FTIR spectroscopy proved that, apart from S groups (C–SH and C=S), GQDs have N groups such as amino and amide groups. Gamma irradiation caused a small increase in the GQD diameter (23, 24 and 27 nm) compared to p-GQDs (14 nm), dots preserved a round shape, they were homogenously dispersed and no agglomerates were observed. The height of dots was lowered and the highest fraction of single and double-layered dots (19.1 and 25.8%, respectively) was detected in the GQDs-cys-200 sample. The ability of dots to generate singlet oxygen upon light illumination was detected only for GQDs-cys-25 and at the same time this sample showed the best visibility of HeLa cells on a fluorescence microscope. According to the MTT assay, gamma-irradiated GQDs were not toxic toward HeLa cells after treating them with concentrations from 1 to 100 μg mL^−1^ during 24 and 48 h. These results led to the conclusion that GQDs-cys-25 is a good candidate for both photodynamic therapy and bioimaging. To further improve the structure of irradiated and modified GQDs for use in biomedicine, our future experiments will be based on this and related studies.

## Figures and Tables

**Table 1:** Positions of the emission band center in nm at excitation wavelength from 300 to 400 nm.

Sample	Position (nm)	Sample	Position (nm)
p-GQDs	426^Ex300^	GQDs-cys-50	440^Ex300^
	439^Ex320^		445^Ex320^
	452^Ex340^		457^Ex340^
	464^Ex360^		465^Ex360^
	477^Ex380^		478^Ex380^
	494^Ex400^		493^Ex400^
GQDs-cys-25	443^Ex300^	GQDs-cys-200	435^Ex300^
	447^Ex320^		442^Ex320^
	457^Ex340^		456^Ex340^
	466^Ex360^		467^Ex360^
	480^Ex380^		478^Ex380^
	495^Ex400^		494^Ex400^

**Table 2:** Values of integrated surface for emission spectra at excitation wavelength 360 nm, absorbance at 360 nm and calculated QY.

Sample	I_ex360_	A_360_	QY (%)
p-GQDs	23,450,791^340nm^	0.144^340nm^	1.45^340nm^ [33]
GQDs-cys-25	35,957,065	0.073	21.60
GQDs-cys-50	17,488,510	0.149	5.15
GQDs-cys-200	14,572,620	0.205	3.12
Rhodamine B	66,440,340	0.094	31

**Table 3:** The values of the average diameter and height of GQD samples, before and after irradiation.

Sample	Diameter (nm)	Height (nm)
p-GQD	20.4	2.6
GQDs-cys-25	25.5	1.6
GQDs-cys-50	28.0	1.5
GQDs-cys-200	29.0	1.4

## Data Availability

Not applicable.
